# The Glucosinolates: A Sulphur Glucoside Family of Mustard Anti-Tumour and Antimicrobial Phytochemicals of Potential Therapeutic Application

**DOI:** 10.3390/biomedicines7030062

**Published:** 2019-08-19

**Authors:** James Melrose

**Affiliations:** 1 Honorary Senior Research Associate, Raymond Purves Bone and Joint Research Laboratory, Kolling Institute of Medical Research, Royal North Shore Hospital, Faculty of Medicine and Health, The University of Sydney, St. Leonards, NSW 2065, Australia; james.melrose@sydney.edu.au; 2 Graduate School of Biomedical Engineering, Faculty of Engineering, University of New South Wales, Sydney, NSW 2052, Australia; 3 Sydney Medical School, Northern, Royal North Shore Hospital, St. Leonards, NSW 2065, Australia

**Keywords:** glucosinolate, sulphopharane, allyl isothiocyanate, phase II detoxification enzymes, anti-tumour agents, anti-bacterials

## Abstract

This study reviewed aspects of the biology of two members of the glucosinolate family, namely sinigrin and glucoraphanin and their anti-tumour and antimicrobial properties. Sinigrin and glucoraphanin are converted by the β-sulphoglucosidase myrosinase or the gut microbiota into their bioactive forms, allyl isothiocyanate (AITC) and sulphoraphanin (SFN) which constitute part of a sophisticated defence system plants developed over several hundred million years of evolution to protect them from parasitic attack from aphids, ticks, bacteria or nematodes. Delivery of these components from consumption of cruciferous vegetables rich in the glucosinolates also delivers many other members of the glucosinolate family so the dietary AITCs and SFN do not act in isolation. In vitro experiments with purified AITC and SFN have demonstrated their therapeutic utility as antimicrobials against a range of clinically important bacteria and fungi. AITC and SFN are as potent as Vancomycin in the treatment of bacteria listed by the World Health Organisation as antibiotic-resistant “priority pathogens” and also act as anti-cancer agents through the induction of phase II antioxidant enzymes which inactivate potential carcinogens. Glucosinolates may be useful in the treatment of biofilms formed on medical implants and catheters by problematic pathogenic bacteria such as *Pseudomonas aeruginosa* and *Staphylococcus aureus* and are potent antimicrobials against a range of clinically important bacteria and fungi. The glucosinolates have also been applied in the prevention of bacterial and fungal spoilage of food products in advanced atmospheric packaging technology which improves the shelf-life of these products.

## 1. Introduction

**Figure 1:**
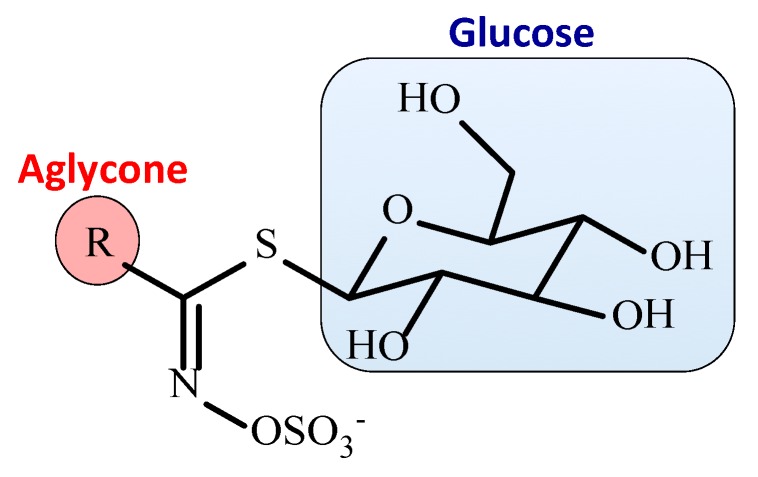
Generic structure of the Glucosinolates showing glucose, sulphation and the aglycone side chain (R) used to categorize the aliphatic, indolic or aromatic glucosinolates.

Plants produce a myriad of phytochemicals and many of these have valuable nutritive, medicinal and health promoting properties [1,2,3] (Table 1, Table 2 and Table 3). Anecdotal evidence often points to these beneficial properties however in this report we will concentrate on two members of the glucosinolates (Figure 1), Glucoraphanin and Sinigrin, with a very extensive scientific and nutritional literature illustrating their potential therapeutic applications [3,4,5,6,7,8,9,10,11,12] (Table 2).

Cruciferous plants such as those listed in Table 1 represent important components of a healthy diet and have characteristic spicy flavor profiles which are appealing to many and have important effects in several physiological processes.

## 2. The Natural Anti-Microbial Activity of Glucosinolate Rich Foods

**Figure 2:**
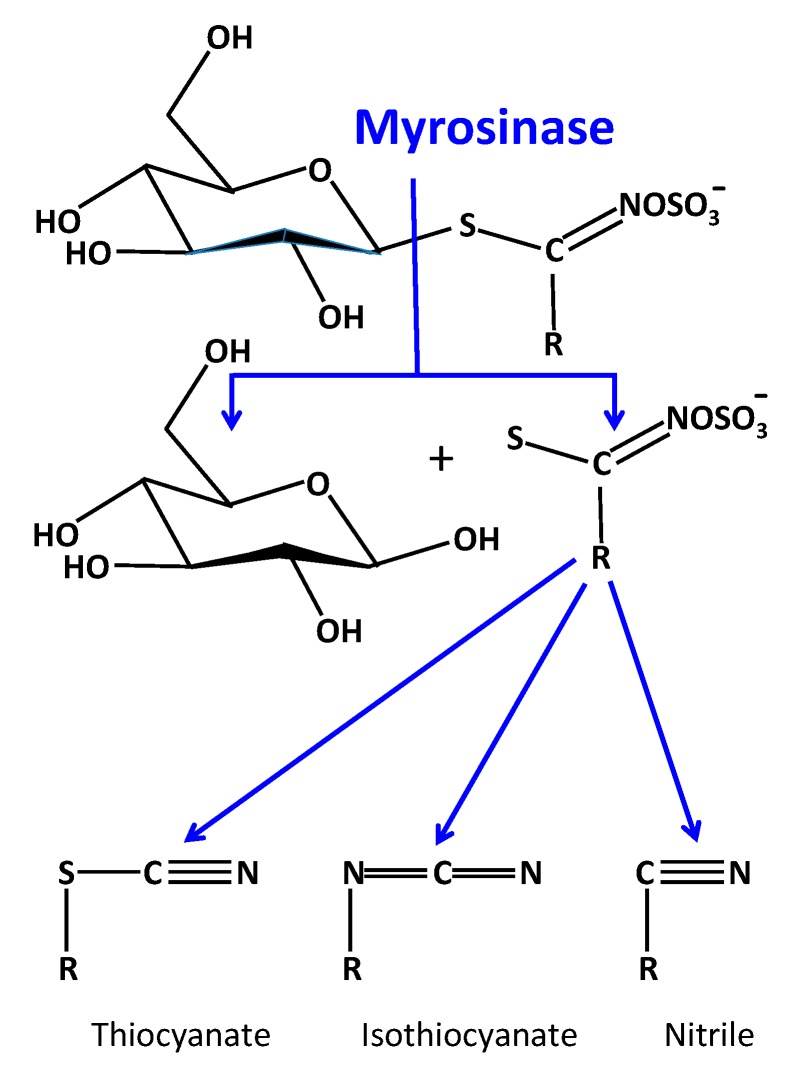
Enzymatic processing of the glucosinolates by myrosinase into bioactive components.

**Figure 3:**
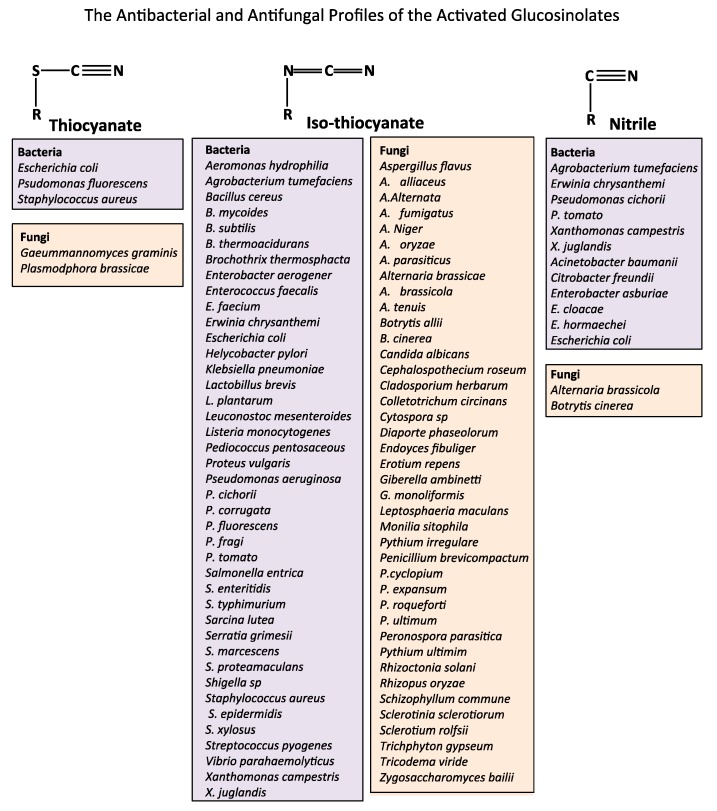
The antimicrobial activities of glucosinolate thiocyanate, iso-thiocyanate and nitrile derivatives.

When plant tissues are damaged, myrosinase, a β-thioglucosidase converts the glucosinolates (Figure 1) to nitriles, thiocyanates and isothiocyanates (Figure 2) which have potent antimicrobial activity with the isothiocyanates in particular displaying potent antibacterial and anti-fungal activity profiles (Figure 3). The inclusion of dietary cruciferous vegetables rich in the glucosinolates may counter antibiotic-resistant bacteria in the food chain arising from the overuse of antibiotics in animal rearing practices. The traditional use of mustard-derived flavoring condiments, while contributing desirable flavor profiles to cooked food items, also provides food preservative properties which traditional societies have relied upon in the prevention of microbial spoilage of foods [13]. This is particularly important in climatic conditions and ambient temperatures conducive to microbial growth leading to food spoilage. Until relatively recently, these societies did not have access to refrigerated storage facilities thus mustard seed products played an important role in food preservation. Mustard seed oil is a potent source of bioactive glucosinolates and represents approximately 30% of the edible oil market in SE Asia. The widespread use of this oil has positively contributed to food storage properties and protection from microbial infection [13,14]. The glucosinolates and their derivatives are volatile compounds and this property has been applied in modern gaseous food packaging technology to extend the shelf-life of food products [14,15]. All cereals have fungal spores associated with the grain surface and the husk, thus whole milled cereal flours used for bread production contain fungal spores. These are inactivated during the baking process; however, fungal spoilage of bread and bakery products can still occur in the post-baking storage and or processing of bakery products. Rape seed oil or mustard flour have been evaluated in bread production to minimize fungal spoilage [16], the major active glucosinolates in rape seed brown mustard (*Brassica juncea*) oil are AITC (85%) and butenyl isothiocyanate (10%) [13] and these have broad fungicidal activity (Figure 3). In the bakery environment, 2 ppm AITC inhibited the growth of *Penicillium roqueforti, P. corylophilum, Eurotium repens, A. flavus and Endomyces fibuliger* on rye bread stored in an airtight environment [17]. Modified atmospheric packaging formats (85% CO_2_, 1% O_2_) combined with mustard oil vapour packaging has been used to extend the storage properties of bread and bakery products [18] and enhances the potency of AITC as a preservative [18,19].

## 3. The Brassicaceae Family of Plants

As already indicated, the Brassicaceae are a rich source of sulphur glucoside glucosinolates, these impart a characteristic spicy flavor profile to these vegetables. Glucosinolates have been classified into three categories on the basis of their amino acid precursors (i) aliphatic (e.g., glucoraphanin; Ala, Leu, Ileu, Val, Met), (ii) indole (e.g., glucobrassicin; Trp), and (iii) aromatic (e.g., gluconasturtiin; Phe, Tyr). While ~130 glucosinolates have been identified to date, in a survey of 2,121 German participants in the European Prospective Investigation into Cancer and Nutrition (EPIC study), only five of these glucosinolates were commonly found in the human diet, glucobrassicin, sinigrin, glucoraphasatin (dehydroerucin), glucoraphanin, and glucoiberin [20].

**Figure 4:**
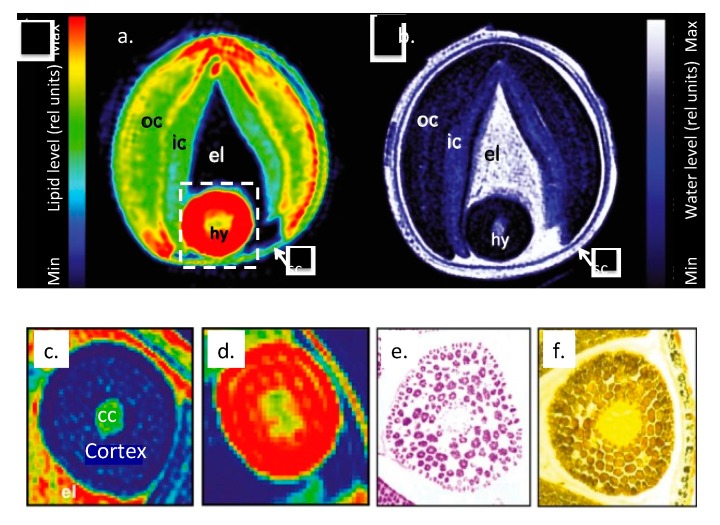
Lipid and moisture storage in *Brassica napus* seeds (**a**,**b**) and hypocotyl (**c**,**d**) visualized by non-invasive MRI. The concentration of water and oil are colour coded red (high); blue, (low). Crucifern immunolocalisation (**e**) and iodine stained starch (**f**) modified from [24] under Creative Commons Deed Attribution licence 2.5. oc,/ic outer/inner cotyledon; el, endosperm; hy, hypocotyl; sc, seed coat; cc, central cylinder.

Glucosinolates have only been found in dicotyledonous plants occurring mainly in the Capparales order (Figure 1 and Figure 2) including cruciferous vegetables and the mustards *Brassica juncea* (brown mustard) [21], *Brassica napus*. (rape seed) and the popular Japanese condiment horseradish Wasabi (*Eutrema japonicum* or *Wasabia japonica*) [22,23] (Table 1). The glucosinolates are stored in a concentrated form in the seed heads and are extracted in cold pressed oils but are also components of the stem and leaves of these plants (Figure 4).

**Figure 5:**
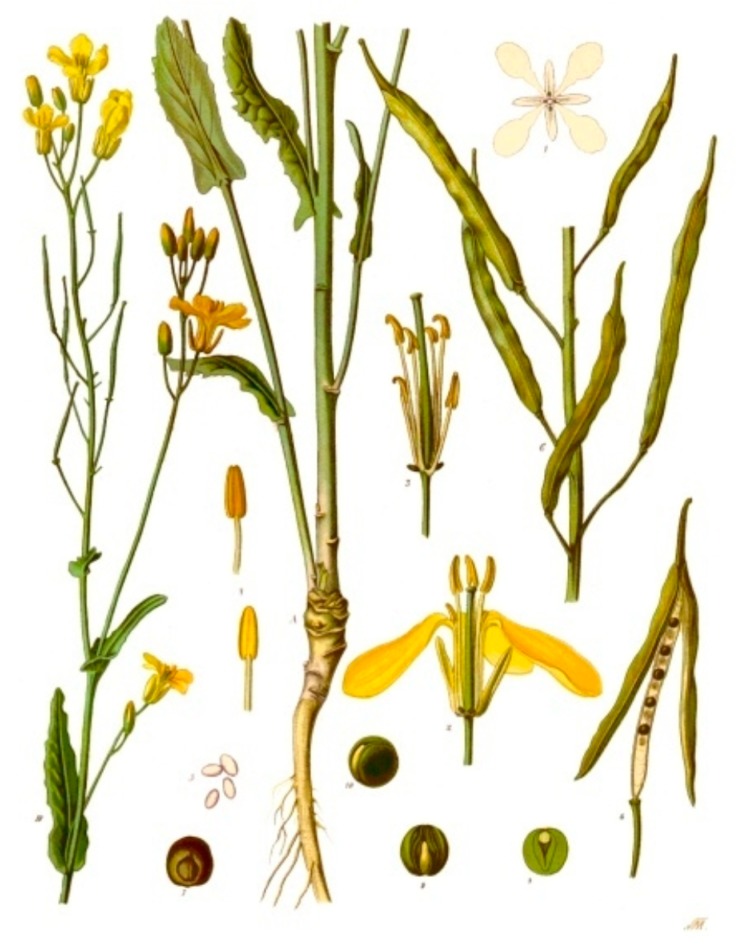
Anatomical description of a mustard (*Brassica napus*) plant showing its characteristic four petal flower head, stamen, seed pods, leaf arrangements and seeds. Image from Franz Eugen Koehler archive, Koehlers Medicinal Plants, Germany 1887. Image reproduced from Wikimedia Commons Repository, https://en.wikipedia.org/wiki/Rapeseed (accessed 19 August 2019), copyright lapsed.

The mustard plant, rapeseed, yellow, white and brown mustards are widely distributed and have a characteristic yellow flower head (Figure 5). Rapeseed (*Brassica napus*), also known as rape, oilseed rape [25] is a member of the *Brassicacea*, mustard or cabbage family named from the Latin word for turnip, *rapum* [26]. This is an ancient plant known of since Biblical times, which has even been identified in the fossil record of the Mesozoic era/mid-Devonian period in Western China. Identification of fossil remains in food cooking implements suggest that mustard seeds may have been the first ever condiment used to flavor food by prehistoric man [27]. Plant evolutionary studies show that the mustard seed plant was of fundamental importance to the subsequent evolution of most other modern day cultivated plants. The leaves, seeds, and roots of wild mustard *Cleome viscosa* have all been widely used in traditional and folkloric medicine for generations. In Ayurvedic medicine mustard was reported to have many beneficial properties, subsequent scientific and pharmacological studies verified it’s antimicrobial, analgesic, anti-inflammatory, antipyretic, anti-diabetic and hepatoprotective qualities [28,29,30,31]. Subsequent studies have identified the phytochemicals responsible for these activities as shown in the present study; glucosinolates are prominently represented on this list of bioactive compounds.

*Brassica napus* was botanically described and published in *Species Plantarum* by Carl Linnaeus, who introduced the binomial name *Brassica napus* for the first time in 1753 [25] (Figure 5).

Rapeseed oil is one of the oldest known vegetable oils, but historically has been used in limited quantities as a food item due to its high levels of erucic acid, natural rapeseed oil can contain up to 54% *w*/*v* erucic acid [32]. Rapeseed cultivated for food production typically contains ~0.5–5% *w*/*v* erucic acid. Erucic acid (C_22_H_42_O_2_) is a C22 chain mono-unsaturated omega-9-fatty acid. A strain of mustard subsequently developed with low erucic acid and glucosinolate levels, Canola, a contraction of the terms “*Canada”* and “*ola”*, is a low erucic acid, low glucosinolate rapeseed [33]. Canola oil is limited by government regulation to a maximum of 2% *w*/*v* erucic acid in the USA and 5% *w*/*v* in the EU. In 1992, the health promoting properties of rapeseed oil gained publicity in the George Miller feature film “Lorenzo’s Oil” starring Nick Nolte and Susan Sarandon, which documented the work of a British chemist, Don Suddaby, and Augusto Odone in 1985 who developed a blend of rapeseed and olive oils which halted the progression of Adrenoleukodystrophy, a genetic disorder characterized by an enzyme abnormality resulting in the build-up of toxic fatty acid levels in the brain damaging the myelin sheaths impairing neuronal function and resulting in convulsions, seizures and hyperactivity. The antioxidant properties of activated glucosinolate compounds are also conducive to the maintenance of brain health [34,35,36,37,38,39,40,41,42,43,44]. The brain is a fatty acid rich tissue and particularly prone to redox ROS mediated mitochondrial damage during neuroinflammation [45,46].

## 4. Public Health Concern over the Impact of Antibiotic-Resistant Bacteria

There is considerable current-day public concern about the overuse of antibiotics in husbandry practice in order to maintain animal health and commercial output levels. The emergence of antibiotic-resistant organisms in humans is related to this agricultural practice. This has been acknowledged by the WHO and by the publication of government guidelines on the use and abuse of antibiotics in agricultural practice. The publication of a list of antibiotic-resistant pathogenic bacteria of particular concern by the WHO (Table 3), and the allocation of major research funds to national agencies in the USA, Canada and Australia to address the problem of antibiotic-resistant bacteria, testifies to the significant threat these organisms represent to human health.

### 4.1. Treatment of Antibiotic-Resistant Bacterial Infections

Antibiotics and antimicrobial medications have been a mainstay in the treatment of infectious diseases for over 70 years and have been an essential component in healthcare practice to combat bacterial and fungal infections. Widespread use of antibiotics and indeed their over-prescription in healthcare circles, plus an overuse of antibiotics in animal rearing practices in agriculture, has led to infectious organisms being widely exposed to these compounds and, as a consequence, this has actually selected for organisms which have developed a resistance to these compounds and these strains of bacteria and fungi now represent a significant healthcare risk on a global scale. An estimated 2 million patients have become infected with antibiotic-resistant bacterial strains in the USA and as a consequence 23,000 deaths were recorded directly arising from these bacterial infections. Multi-drug-resistant bacterial infections were also responsible for an estimated 25,000 deaths per year in the EEC in 2015–2017 and these cost €1.5 billion per year in healthcare treatment and lost productivity. If these current infection rates are not reversed then 10 million deaths globally per year are predicted by 2050 (317,000 in the USA; 392,000 in S. America; 392,000 in EEC; 4.1 million in Africa; 4.7 million in Asia and 22,000 in Australia). Moreover, it is estimated that additional hospital costs per patient will be in the order of 10,000–40,000 $US in OECD countries. Furthermore, the associated impact of lost economic output due to increased mortality, prolonged sickness and reduced labour efficiency may effectively double this figure. In vitro studies on the activated thiocyanates, isothiocyanates and nitrile compounds generated from the glucosinolates by myrosinase demonstrate these are suitable compounds for antibacterial and anti-fungal evaluations in the treatment of such infections (Figure 3). Furthermore, some of these plant compounds synergise with existing antibiotic treatment protocols (gentamycin, vancomycin) and may represent a useful adjunct to these treatments [47]. *Listeria monocytogenes* and *Staphylococcus aureus* in particular were significantly inhibited by benzylisothiocyanate and 2-phenylethylisothiocyanate in isolation or in phytochemical-antibiotic combinations.

### 4.2. Commercial Development of Antibiotics

Antimicrobial resistance is a global crisis that threatens the public healthcare system. Development of novel antibiotic products is a critical component to combatting antimicrobial infections [48]. A survey of all participants and interested parties in antibiotic research in 2015 in the European Union was undertaken to develop new economic incentives to stimulate greater antibacterial drug innovation [48]. Unfortunately, the financial incentives to undertake such research have proven to be insufficient for many major pharmaceutical companies to maintain investment in antibiotic research programs. The announcement by Novartis of its intention to exit from all antibiotic research in 2018 joined AstraZeneca, Sanofi, and Allergan who also exited from this type of research due to the high cost of undertaking such research coupled with a lack of financial return. This leaves Merck, Roche, GlaxoSmithKline, and Pfizer as the remaining pharmaceutical companies that have continuing active antibiotic research programs. An editorial in Nature Biotechnology in 2018 entitled “Wanted: a reward for antibiotic development” summarised findings of the Nature conference “Countering Antimicrobial Resistance” held in Beijing, China in 2018 which showcased a biodiverse array of discovery approaches currently being undertaken globally to combat drug-resistant bacteria [49]. Even so, development of new antibiotics has dwindled to dangerously low levels in the past three decades. It is well recognised that this lack of innovation has perilous consequences on available treatments for pathogenic infections to the detriment of patient care. This has resulted in the formation of several government agencies and collaborative platforms to support the discovery of new antibiotics.

The *European Observatory on Health Systems and Policy* has been assembled to support the development of new therapeutic antibiotics and contains members from The European WHO Regional Office, European Governments (Austria, Belgium, Finland, Ireland, Norway, Slovenia, Sweden, the UK, Veneto Region in Italy), European Commission, World Bank, National Union of Health Insurance Funds in France, Schools of Economics and Political Science; Hygiene and Tropical Medicine in London, UK. The Observatory has a secretariat in Brussels and hubs in London and Technical University of Berlin. A 133-page report issued in 2016 by Renwick, Simpkin and Mossialos entitled “Targeting innovation in antibiotic drug discovery and development: The need for a One Health – One Europe – One World Framework” [50] recommends several initiatives to promote antibiotic research including financial incentives, R&D support, effective coordination and dissemination of findings from European agencies, effective collaboration with outside agencies and preclinical support [50].

The pharmaceutical industry has not released any new antibiotic formulations for over three decades, and this has resulted in an alarming incidence of deaths resulting directly from antibiotic-resistant bacteria. The World Health Organisation (WHO) has publicised this as a major public healthcare issue, indicating the real possibility that without new antibiotic treatments becoming available, we may be entering an era when even previously treatable bacterial infections will become life-threatening. The repurposing of anti-cancer drugs for the treatment of bacterial infections has been suggested since some of these have proven to be effective in vitro for the elimination of recalcitrant, multidrug tolerant bacteria, while other antibiotics are useful as anti-cancer compounds [51,52,53,54]. Among the most harmful human pathogenic bacteria, *Staphylococcus aureus (Golden Staph)* stands out as one of the most virulent and troublesome due to its ability to cause life-threatening infections and to readily adapt to changing environmental conditions [55,56]. The ability of *S.aureus* to establish itself in various community home and hospital environments, and its resistance to antibiotic treatment make this an important healthcare threat [57]. The emergence of methicillin resistant *S.aureus* (MRSA) almost five decades ago demonstrates the serious nature of such infections. Hospital environments are conducive to *S.aureus* colonisation and its virulence is a major threat particularly to patients with reduced immune function. Particularly virulent strains of *Enterococcus*, resistant to conventional antibiotic treatment, have also emerged in hospitalized patients [58]. Of particular concern are the vancomycin-resistant enterococci (VRE), that lead to infections of the urinary tract associated with prolonged catheter use or to catheter mediated bloodstream infections [59]. There is therefore an increasing global interest in the identification of bioactive compounds from plant sources, which display antibacterial and anti-fungal properties that are pharmacologically effective but which display limited or no side effects. The glucosinolates produced by the *Brassicacea* family, order Capparales contain compounds with potent antibacterial, anti-fungal, anti-nematodicidal, anti-viral and insecticidal properties making them obvious candidates in the search for compounds to counter bacterial infections [4,10,11,60,61,62,63,64,65,66]. Morever, many of the glucosinolates act synergistically with existing antibiotic regimens improving their effectiveness [47,63]. A list of antibiotic-resistant “priority pathogens” published by WHO in 2017 covers 12 bacterial families posing the greatest threat to human health [67] and highlights Gram-negative bacteria resistant to multiple antibiotics which threaten global public health, these have been referred to as Super-bugs [68,69,70].

The effective antibiotics available for the treatment of bacterial infections are relatively small in number and in many cases have become largely ineffective. The last time a new antibiotic was released on to the world market was approximately 30 years ago, there is a strong need for antibiotic development and a world market eagerly awaiting this product. The WHO has established three treatment categories based on the urgency for new antibiotics: these are critical, high and medium priority (Table 3). The WHO have categorized critical, high, and medium priority treatment areas for antibiotic-resistant bacterial strains (Table 3) and have identified hospital and nursing home patients with reduced immune function as being a particularly susceptible group to such infections. Patients who regularly use ventilators, dialysis machines and medications requiring long-term administration by catheter are liable to become infected with strains of *Acinetobacter*, *Pseudomonas*, *Klebsiella*, *E. coli*, *Serratia*, and *Proteus* that are capable of causing severe and often deadly bloodstream infections and the development of pneumonia. Unfortunately, bacterial strains have emerged which are no longer responsive to the carbapanem and third generation cephalosporins, which were previously the most effective compounds used to treat such infections. Gonorrhoea is rapidly becoming a condition which will soon become untreatable.

The common practice of routine administration of broad-spectrum antibiotics to treat an infection before identification of the specific pathogen responsible for an infection has proven to be an ill-advised practice. By removing the normal bacterial microflora, antibiotics actually provide an opportunistic niche for the emergence of antibiotic-resistant bacterial strains which no longer have to compete with the normal bacterial populations present in the body [71,72]. *Klebsiella pneumoniae* is a Gram negative, facultative anaerobic commensal microorganism that can cause chronic urinary tract and soft tissue infections, pneumonia, and sepsis, and mostly occurs in immunocompromised patients [73]. *Klebsiella pneumoniae* normally colonises the mouth, skin, and intestines. Illness predominantly affects middle-aged and older men with debilitating diseases but infants are increasingly now being reported with this organism in urinary tract and intestinal infections [74,75,76]. The emergence of multi-drug-resistant bacterial infections in hospitalized patients with underlying morbidity is of particular concern [77]. In a recent US epidemiology study [72] 25% of *K. pneumoniae* infections in long-term acute care hospitals were resistant to carbapenems, which are currently used to treat penicillin-resistant Gram-negative pathogens. The most common condition caused by *Klebsiella* bacteria outside the hospital environment is pneumonia, bronchopneumonia and bronchitis and has a death rate around 50%, even when antimicrobial therapy is administered. Gram negative Enterobacteriaceae bacteria such as Klebsiella have evolved β-lactamase genes which counter the effectiveness of carbapanem as an antibiotic and have resulted in the rapid spread of such antibiotic-resistant bacteria worldwide [78]. Transmissible carbapenem-resistant Enterobacteriaceae have been recognised for the last two decades; however, the global dissemination of these bacteria is a more recent pandemic and is now recognised to be occurring at an alarming pace [79]. Identification of Klebsiella pneumoniae carbapenamase-producing K. pneumonia as a deadly pathogen is of particular concern due to the rise of its global incidence in pediatric and neonatal intensive care units [80,81,82].

## 5. WHO, United Nation and World Bank Programmes and Coordinated Interagency Collaborations Designed to Combat Antibiotic-Resistant Bacteria

A number of National and International Government initiated and private sector organisations have emerged which aim to investigate better methods to combat the threat of drug-resistant bacterial strains. Stated operational areas, research objectives and areas of expertise for each organisation demonstrate a diverse approach to combating this global public health concern and some areas of overlap, however in order to avoid duplication, redundancy and areas of wasted effort further agencies have also been set up to co-ordinate and disseminate research findings to all interested parties. Independent private commercial organisations are also actively engaged in the search for effective treatments for antibiotic-resistant organisms to service an immense global market.

The Global Antimicrobial Resistance Surveillance System (GLASS) is a WHO initiative which was established to support a systematic approach to the collection, analysis and dissemination of antimicrobial resistance data at a global level to facilitate informed decision-making, at the local, national and regional action areas. GARDP, The Global Antibiotic Research and Development Partnership was also formed by WHO and DNDi, Drugs for Neglected Diseases initiative. GARDP undertakes research and development through public-private partnerships. IACG, Interagency Coordination Group on Antimicrobial Resistance is an initiative of the United Nations Secretary-General which was established to improve coordinated efforts between international organisations and to ensure effective global health security activity [83].

CARB-X has indicated its intention to release up to four new antibacterial treatments based on modifications of current antibiotics in the next 4 years. The UN Deputy Secretary-General and WHO Director General are co-chairs on IACG along with executive members from several other UN and international agencies and acknowledged experts from several industry sectors.

The Centers for Disease Control and Prevention (CDC) and related US agencies are also actively involved in several measures to combat antibiotic-resistant bacterial infections through a collaborative global approach across all government and private sector agencies. CDC has published “CDC. The Core Elements of Human Antibiotic Stewardship Programs in Resource -Limited Settings: National and Hospital Levels. Atlanta, GA: US Department of Health and Human Services, CDC; 2018. Available at: https://www.cdc.gov/antibiotic-use/healthcare/implementation.html” (accessed 14 July 2019) to help improve guidelines for antibiotic use in healthcare settings worldwide. The Food and Drug Administration (FDA) has also announced plans to combat antibiotic resistance through innovative antibiotic developments and the coordinated use of antibiotics in human medicine and in animal husbandry practice. CARB-X, a global non-profit partnership launched in 2016, is dedicated to accelerating antibacterial research to counter the global impact of drug-resistant bacteria This organisation, led by Boston University is currently funding 33 projects in N. America, Europe and Asia. CARB-X is funded by BARDA, The US Department of Health and Human Services Biomedical Advanced Research and Development Authority which is part of the Office of the Assistant Secretary for Preparedness and Response (ASPR). Other organisations which form part of the CARB-X initiative include The Wellcome Trust, a global UK based charity working to improve global health. BMBF, Germany’s Federal Ministry of Education and Research and the United Kingdom, The Global Antimicrobial Resistance Innovation Fund based in the United Kingdom (UK GAMRIF), and world’s largest humanitarian Foundation (Bill and Melinda Gates Foundation) also contribute funding to CARB-X and National Institute of Allergy and Infectious Diseases (NIAID) and US National Institutes of Health (NIH) also provide in-kind support. CARB-X has reported that they will invest >$500 US million by 2021 into the development of antibiotics to combat the deadliest super-bugs, and develop vaccines, rapid diagnostics, and other life-saving products to aid in the treatment of antibiotic-resistant bacteria. This supports *The US National Action Plan for Combatting Antibiotic-Resistant Bacteria*, AR: https://www.cdc.gov/DrugResistance/us-activities.html. (accessed 2 July 2019) Strategies being developed in Australia to combat bacterial resistant infections involve a unified approach by all government and private agencies to combat the threat of antibiotic overuse and development of antibiotic-resistant bacterial infections. 

## 6. Application of the Myrosinase-Glucosinolate System in Biomedicine

The bioactivity of glucosinolate hydrolysis products and potential biomedical applications are well documented (Table 3, Table 4, Table 5 and Table 6). SFN has roles in cancer prevention, high blood pressure, macular degeneration and stomach ulcers and is a potent inducer of mammalian phase II detoxication enzyme systems which deactivate and excrete many carcinogens. The induction of NAD(P)H quinone reductase, heme oxygenase 1 (HO-1), glutamate-cysteine ligase catalytic subunit, and glutathione S transferases occurs through the Keap1-Nrf2-ARE cell signaling pathway [84,85,86]. Numerous studies in human colon, leukemia, pancreatic, lung, and skin cancer cell lines have demonstrated SFN’s inhibitory effects on cell cycle arrest [12,87,88,89] and elevated apoptosis in human bladder [90] and prostate [91] cell lines. Sulforaphane’s ability to disrupt tubulin and actin polymerization, inhibits mitotic spindle formation and tumour cell growth in animal models of breast cancer [92,93] and also inhibits histone deacetylase, increasing apoptosis in human colon, prostate, and kidney cell lines [94,95,96,97].

### 6.1. The Bioactivity of Glucosinolates

The glucosinolates are benign molecules requiring conversion by myrosinase to bioactive thiocyanate, isothiocyanate and nitrile derivatives (Figure 2). Therefore, glucoraphanin and sinigrin are converted into bioactive SFN and AITCs with fungicidal, bactericidal, nematocidal, antioxidant and anti-cancer properties. Biofilm formation on medical devices and implants such as catheters, mechanical heart valves, pacemakers, prosthetic joints, and contact lenses pose a critical medical problem. The most common biofilm-forming bacteria include *Enterococcus faecalis*, *Staphylococcus aureus*, *Staphylococcus epidermidis*, *Streptococcus viridans*, *Escherichia coli*, *Klebsiella pneumoniae*, *Proteus mirabilis*, and *Pseudomonas aeruginosa* [98,99,100,101,102,103], *S. aureus* and *S. epidermidis* are most commonly found on cardiovascular devices [104,105,106], it estimated that 40%–50% of prosthetic heart valve infections, and 50%–70% of catheter biofilm infections are due to these bacteria [107,108]. Despite the evaluation of a wide range of anti-fouling compounds [103,109,110] improvements are still required in this area. Glucosinolates have proven useful in the prevention of biofilm development by *Pseudomonas aeruginosa* [5,111,112,113].

Cooking of cruciferous vegetables inactivates myrosinase activity however the gut microbiota in humans may provide myrosinase activity and lead to absorption of SFN and AITCs in the intestine. A diet rich in cruciferous vegetables is associated with a lower risk of developing breast, lung, prostate, and colorectal cancer [114,115,116,117,118]. It is important to control the redox balance in the human brain to control neuronal mitochondrial activity, oxidant stress on mitochondria can diminish neuronal energetics and promote neurodegeneration in Parkinson’s and Alzheimers’s disease [119]. Brain tissue is very rich in fatty acids and is especially sensitive to the action of free radical oxidant activity. The GSTs are ROS scavengers and are neuroprotective [119,120,121].

### 6.2. Cancer and Dietary SFN and AITC Levels

Meta analyses of clinical trials on dietary glucosinolates have generally provided promising but not compelling evidence of the efficacy of these as anti-oxidants or anti-cancer agents despite positive in vitro findings in cell culturing experiments and may reflect the inefficiencies of the dietary route for delivery of these compounds. Positive effects are generally achieved in vitro with concentrations of the active glucosinolate components in the 1–40 µmol range. It is unlikely that this level of therapeutic agent would be delivered successfully to the target tumour cells in vivo by the diet. Attempts have been made to increase the glucosinolate content of broccoli hybrids, broccoli sprouts are also richer sources of the glucosinolates, particularly since these are consumed uncooked thus endogenous myrosinase is not inactivated by the cooking process and it has time to convert the glucosinolates to bioactive forms during food mastication. The detection of SFN and AITCs excreted in urine and faecal matter following consumption of cooked cruciferous vegetables where the endogenous myrosinase is inactivated in the initial cooking stages, indicates that the gut microbiota are another source of myrosinase activity. Therefore, therapeutic doses of SFN and AITCs are likely achievable to target tumour cells in the colon [104,210,211], prostate [91,95,171,172,173] and bladder [162,188,189,190,191,192,193]. Dietary glucosinolates are also effective in the treatment of gastric *H.Pylori* infections and gastric cancer. The delivery of therapeutic doses of dietary SFN and AITCs through the systemic circulation to pancreatic, ovarian, breast and liver cancer and melanoma, however, is less likely to be as effective and may explain the relatively poor findings of meta analyses of dietary clinical trials on the glucosinolates as anti-cancer agents. In many cases, the statistical power achieved in these analyses has also been reduced by low sample sizes or no associations were established. More high-quality cohort studies with larger sample sizes and well controlled confounding factors are required to confirm the benefit of dietary cruciferous vegetable consumption; initial studies have delivered sufficient evidence to warrant such studies. The bioavailability of glucosinolates following different food processing methods has also been evaluated in order to improve the dietary content of bioactive forms of the glucosinolates [217] Supplementation of the diet with broccoli sprouts or myrosinase containing mustard products have also been examined as a means of increasing the dietary SFN and AITC content [218]. The effective delivery of SFN and AITCs to the target cells in solid tumours is a difficult proposition. Delivery systems based on hyaluronan as a carrier molecule have been developed for several steroids and cytotoxic compounds and successfully treated solid tumours however this methodology has yet to be applied to the delivery of SFN or AITCs in these problematic cancers (reviewed in [219]).

### 6.3. The Beneficial Bioactivities of Sinigrin and Their Applications in Biomedicine

Although the scientific literature on sinigrin (Table 7) is not as extensive as that of SFN, they share similar bioactivities and areas of application and if supplied as a dietary component will not be acting in isolation anyway [113].

## 7. Concluding Remarks

The myrosinase-glucosinolate system in plants is a sophisticated protective system that developed over several hundred million years of evolution. With a greater understanding of the system’s component parts, it is now possible to apply this knowledge to human physiological processes, an advance that is of potential benefit in biomedicine. Some of these compounds may be useful in the prevention of fouling of plant equipment, sterilisation of medical implants, wound healing and the prevention of some forms of cancer. The extensive literature documenting the biodiversity of glucosinolate applications in biomedicine indicates considerable promise in future areas of investigation in:

1. Antibiotics, anti-fungal and anti-viral agents
2. Biofilm prevention in medical implants, catheters and industrial plant equipment
3. Nutritive additives with anti-cancer properties
4. Advanced food packaging technology to improve shelf-life of food products.

## Figures and Tables

**Table 1:** Examples of Glucosinolate rich Cruciferous plants of the *Brassicacea* family order Capparales.

Plant Types
Brocolli
Brocolli Sprouts
Cabbage
Brussell Sprouts
Cauliflower
Daikon (Japanese radish)
Daikon sprouts
Garden Cress (*Lepidum sativum*)
Kale
Rapeseed (*Brassica napus*)
Wasabi (*Wasabia japonica*)
White Mustard (*Sinapis alba*)
Yellow Mustard (*Brassica juncea*)
Bok Choi
Arugula, Rocket (*Eruca sativa*)
Collard Greens
Horseradish
Kohlrabi
Radish
Rutabaga/turnip
Watercress
Mustard Greens

**Table 2:** Aliphatic (**A**), Indolic (**B**) and Aromatic (**C**) Glucosinolates and their contents in *Brassica* vegetables *Capparales* Order (μmol/100 g wet wt tissue) (data modified from [24,25,26]).

Glucosinolate Trivial Name	Aglycone Chemical Name	Aglycone Structure	Vegetable Source	Glucosinolate Content μmol/100 g)
**A. Aliphatic**				
Glucoibervirin	3-Methylthiopropyl	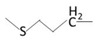	Green Cauliflower White Cauliflower	0–11.8 1.5–7.1
Glucoerucin	4-Methylthiobutyl	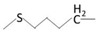	Rocket	52–109
Glucoiberin	3-Methylsulfinylbutyl	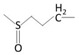	Brocolli Sprouts Savoy Cabbage	59–181 24–50
Glucoraphanin	4-Methylsulfinylbutyl	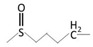	Brocolli Sprouts Brocolli	233–676 24–285
Sinigrin	Prop-2-enyl	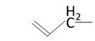	Brussels Sprouts White Cauliflower	46–91 57–121
Gluconapin	But-2-enyl	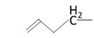	Pak Choi	24–157
Glucovrassicanapin	Pent-2-enyl	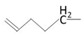	Chinese Cabbage Pak Choi	2.3–25 27–69
Progoitrin	(2R)-Hydroxybut-3-enyl	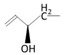	Turnip Chinese Brocolli	18–41 49
**B. Indolic**				
Glucobrassicin	Indol-3-ylmethyl	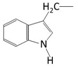	Brocolli White Cauliflower	13–29 11–33
4-Hydroxy-Glucobrassicin	4-Hydroxy-3-indolylmethyl	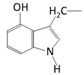	Brocolli White Cauliflower	0.1–3.3 0.2–2.8
4-Methoxy–Glucobrassicin	4-Methoxy-3-indolylmethyl	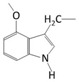	Brocolli White Cauliflower	0.9–2.8 0.7–3.2
Neo-Glucobrassicin	4-Methoxyindol-3-ylmethyl	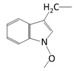	Brocolli White Cauliflower	1.8–13 0.9–3.0
**C. Aromatic**				
Glucotropaeolin	Benzyl	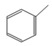	Garden Cress	NA
Gluconasturtiin	Penylethyl	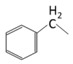	Water Cress	NA

**Table 3:** The WHO Dirty Dozen Pathogen List of Problematic Super Bugs *.

Priority Category	Bacterium	Drug Resistance
Critical	*Acinetobacter baumannii*	
	*Pseudomonas aeruginosa*	Carbapanem
	ESBL** producing members of the *Enterobacteriaceae*	
High	*Enterococcus faecium*	Vancomycin
	*Staphylococcus aureus*	Methicillin/Vancomycin
	*Helicobacter pylori*	Clathrimycin
	*Campylobacter spp*	Fluoroquinolone
	*Salmonellae*	Fluoroquinolone
	*Neisseria gonorrhoeae*	Cepalosporin/Fluoriquinolone
Medium	*Streptococcus pneumonia*	Penicillin
	*Haemophilus influenzae*	Ampicillin
	*Shigella spp*	Fluoriquinolone

* http://www.who.int/news-room/detail/27-02-2017-who-publishes-list-of-bacteria-for-which-new-antibiotics-are-urgently-needed (accessed 12 January 2018); ** Certain strains of bacteria are resistant to treatments with commonly used antibiotics such as penicillin and cephalosporins. These bacteria produce enzymes known as Extended Spectrum Beta-Lactamases (ESBL). ESBL producing bacteria are resistant to most types of third generation antibiotics and include strains of *Klebsiella pneumoniae, Klebsiella oxytoca and Escherichia coli. Enterobacter* spp., *Salmonella* spp., *Morganella morganii*, *Proteus mirabilis*, *Serratia marcescens* and *Pseudomonas aeruginosa* produce ESBLs relatively infrequently.

**Table 4:** Combination Therapies of Sulphoraphane and Conventional Anti-Cancer and Antibacterial Drugs.

Compound Used in Combination Therapy	Reference
SFN-Selenium nanoparticles	[122]
Paclitaxel	[9]
Cisplatin	[123]
Luteolin	[124]
Clofarabine	[125]
Doxorubicin	[126]
5-fluorouracil	[127]
HistoneH3	[128]
Withaferin A	[129]
Hispidulin	[130]
Carboplatin	[131]
Docetaxel	[132]
Lapatinib	[133]
PR-104A	[134]

**Table 5:** Some Examples of The Diverse Therapeutic Applications of Sulphoraphane.

Medical Conditions Treated with Sulphoraphane	
Spatial learning and memory dysfunction	[135]
Chemotherapy-induced neuropathic pain	[136]
Protection of granulosa cells against oxidative stress	[137]
Cadmium-mediated carcinogenesis	[138]
Oxidative stress in cultured adult cardiomyocytes	[139]
Protective effects of glucosinolate hydrolysis products in neurodegenerative diseases	[140]
Clearance of Amyloid-β and Tau protein in a mouse model of AD	[141]
Experimental diabetic peripheral neuropathy	[142]
Joint inflammation in a murine adjuvant-induced mono-arthritis	[143]
Protection against cognitive impairment in AD-like lesions in diabetes	[144]
Anti-inflammatory effect of SFN on human THP-1 macrophages in a murine AD model	[145]
Inhibition of oxidative stress/inflammation improves cardiac function in a Rabbit Model of Chronic Heart Failure	[146]
Inhibition of class IIa histone deacetylase activity	[147]
Apoptosis via microtubule disruption in cancer	[148]
Inhibition of LPS-Induced Inflammation/cytotoxicity/oxidative microglial stress	[149]
Down-regulation of MAPK/NF-κB signaling in LPS-activated BV-2 microglia	[150]
Inhibition of oxidative stress in an in vitro model of age-related macular degeneration	[151]
Modification of Histone H3, unpacking of chromatin, to prime defence	[128]
Modulation of oxidative stress and inflammation in rats with toxic hepatitis	[152]
Modulation of oxidative damage in lead exposed rat hippocampus	[153]
Prevention of dexamethasone-induced myotube atrophy via Akt/Foxo1	[154]
Induction of p53 deficient SW480 cell apoptosis by ROS MAPK signaling	[155]
Role of microRNAs in the chemopreventive activity of SFN	[156]
Novel phosphonate analogs of SFN with in vitro and in vivo anti-cancer activity	[157]
Gastrointestinal protection against *H. pylori* and NSAID-Induced Oxidative Stress	[158]
Protection against sodium valproate-induced acute liver injury	[159]
Enhanced SFN cardioprotection against oxidative stress by 17β-Estradiol	[159]
Photoprotective Effects of SFN and Hispidulin	[130]
Improvement of neuronal mitochondrial function in brain tissue	[160]
Chemoprevention of oxidative stress-associated with oral carcinogenesis	[161]
Amelioration of bladder dysfunction via activation of Nrf2-ARE Pathway	[162]
Protection against aortic complications in diabetes	[163]
Anti-inflammatory effect against amyloid-β peptide via STAT-1 dephosphorylation and activation of Nrf2/HO-1	[164]

**Table 6:** Sulphoraphane Applications in Cancer Models.

Cancer Type	Reference
Leukemia	[87,165,166,167,168,169,170]
Prostate cancer	[91,95,171,172,173]
non-small cell lung cancer cells	[131,174,175]
Pancreatic cancer	[176,177,178,179]
Breast cancer	[92,93,125,126,127,129,132,133,180,181,182,183,184,185,186,187]
Bladder cancer	[162,188,189,190,191,192,193]
Ovarian cancer	[123]
HepG2 Carcinoma Cells	[194,195,196,197,198]
Gastric cancer	[199,200]
Squamous cell carcinoma	[201,202]
Nasopharangeal cancer	[203]
Melanoma	[204]
Glioma	[163,205,206,207]
Colon cancer	[134,208,209]
Lung cancer	[210,211]
Schwannoma	[212]
Colorectal cancer	[213]
Cervical cancer	[214]
Oral cancer	[215,216]

**Table 7:** The Varied Applications of Sinigrin in Biomedicine.

Application	Reference
Reduction of liver fibrosis	[220]
Suppression of NF-κB/MAPK and NLRP3 inflammasome activation in macrophages	[221]
Promotion of wound healing	[113,222]
Anti-cancer properties in methyl glyoxal modification	[223]
Anti-proliferative activity on carcinogen-induced hepatotoxicity	[224]
Biofumigation of potato cyst nematode	[21]
Inhibition of Listeria monocytogenes on bologna sausages	[112]
inhibition of invasion, migration, MMP-2/-9 activities in SK-Hep 1 human hepatoma cells	[225]
Brussel sprout juice mediated effects on cell cycle and adhesion of human colorectal carcinoma cells (HT29) in vitro	[226]
AITC mediated mitotic block, loss of cell adhesion/disrupted cytoskeleton in HT29 cells	[227]
Cytotoxicity and genotoxicity of allyl and phenethyl isothiocyanates, glucosinolates, sinigrin and gluconasturtiin	[228]
Inhibition of microbial growth	[47,65,229]
Effects of dietary sinigrin or indole-3-carbinol on O6-methylguanine-DNA-transmethylase activity and 4-(methylnitrosamino)-1-(3-pyridyl)-1-butanone-induced DNA methylation and tumorigenicity in F344 rats	[230]

## Abbreviations

AD	Alzheimer’s disease
AKT	A serine/threonine-specific protein kinase
ARE	Antioxidant response element
EPA	Environment Protection Agency
ESBL	Extended Spectrum Beta-Lactamases
Keap-1-Nrf2-ARE	Kelch-like erythroid cell-derived protein with CNC homology (ECH)-associated protein 1–anti-oxidant response element
AITC	Allyl isothiocyanate
GARDP	Global Antibiotic Research and Development Partnership
GSK	Glycogen Synthase Kinase
GST	Glutathione-S-transferase
DNDI	Drugs for Neglected Diseases initiative
IACG	Interagency Coordination Group on Antimicrobial Resistance
LPS	Lipopolysaccharide
MAPK	A mitogen-activated protein kinase
NFκB	Nuclear factor kappa light chain enhancer of activated B cells
NLRPR3	Nucleotide-binding domain and leucine-rich repeat–containing protein 3
NSAID	Non-Steroidal anti-inflammatory
PDGF	Platelet derived growth factor
ROS	Reactive oxygen species
SMC	Smooth muscle cell
TNF	Tumour necrosis factor-alpha
